# Effect of route of delivery on heterologous protection against HCV induced by an adenovirus vector carrying HCV structural genes

**DOI:** 10.1186/1743-422X-8-506

**Published:** 2011-11-04

**Authors:** Jie Guan, Bo Wen, Yao Deng, Ke Zhang, Hong Chen, Xiaobing Wu, Li Ruan, Wenjie Tan

**Affiliations:** 1 State Key Laboratory for Molecular Virology and Genetic Engineering, National Institute for Viral Disease Control and Prevention, Chinese Centre for Disease Control and Prevention, Changbai Road 155, Changping District, Beijing 102206, People's Republic of China; 2 College of Life Science, Jilin University, Chang Chun130012, Jilin Province, People's Republic of China

## Abstract

**Background:**

An effective vaccine and new therapeutic methods for hepatitis C virus (HCV) are needed, and a potent HCV vaccine must induce robust and sustained cellular-mediated immunity (CMI). Research has indicated that adenoviral and vaccinia vectors may have the ability to elicit strong B and T cell immune responses to target antigens.

**Results:**

A recombinant replication-defective adenovirus serotype 5 (rAd5) vector, rAd5-CE1E2, and a recombinant Tian Tan vaccinia vector, rTTV-CE1E2, were constructed to express the HCV CE1E2 gene (1-746 amino acid HCV 1b subtype). Mice were prime-immunised with rAd5-CE1E2 delivered via intramuscular injection (i.m.), intranasal injection (i.n.), or intradermal injection (i.d.) and boosted using a different combination of injection routes. CMI was evaluated via IFN-γ ELISPOT and ICS 2 weeks after immunisation, or 16 weeks after boost for long-term responses. The humoral response was analysed by ELISA. With the exception of priming by i.n. injection, a robust CMI response against multiple HCV antigens (core, E1, E2) was elicited and remained at a high level for a long period (16 weeks post-vaccination) in mice. However, i.n. priming elicited the highest anti-core antibody levels. Priming with i.d. rAd5-CE1E2 and boosting with i.d. rTTV-CE1E2 carried out simultaneously enhanced CMI and the humoral immune response, compared to the homologous rAd5-CE1E2 immune groups. All regimens demonstrated equivalent cross-protective potency in a heterologous surrogate challenge assay based on a recombinant HCV (JFH1, 2a) vaccinia virus.

**Conclusions:**

Our data suggest that a rAd5-CE1E2-based HCV vaccine would be capable of eliciting an effective immune response and cross-protection. These findings have important implications for the development of T cell-based HCV vaccine candidates.

## Background

Hepatitis C virus (HCV) is one of the major agents of acute and chronic hepatitis worldwide [1,2]. Around 80% of HCV infections progress to chronic hepatitis. In turn, chronic hepatitis C infection frequently progresses to cirrhosis, and a significant proportion of patients with liver cirrhosis will develop hepatocellular carcinoma (HCC) [3]. Treatment of chronic hepatitis C with interferon alpha and ribavirin is effective in less than 50% of cases [4,5]. Considerable effort has been directed toward development of a safe and effective HCV vaccine, but without any significant clinical success [6]. Thus the development of such a vaccine is vital [7].

A key feature of most vaccines is induction of neutralising antibodies. The genetic variability of HCV is enormous; the site of greatest variability is within the E2 envelope glycoprotein (hypervariable region 1), a major target of neutralising antibodies [8]. Studies in both humans and chimpanzees have yet to demonstrate a clear humoral immune correlation with viral clearance [9-11]. In contrast, some investigations have suggested that strong HCV-specific cytotoxic T cell (CTL) responses are likely to be important in viral clearance and possibly protection [10-19]. Viral persistence is associated with a weak and dysfunctional virus-specific T cell response [15-17]. Studies have indicated that control of an acute HCV infection is associated with a vigorous, broadly-directed, and sustained activation of HCV-specific T cells [9-11,18]. Therefore, engineering an efficient adaptive immune response, especially a T cell response, should be the goal of any HCV vaccine strategy [1,6,7].

At present, little is known about the association between the structural protein (C/E1/E2)-specific T cell responses induced by different immunisation strategies and the accompanying antiviral protection [1,15,19]. We hypothesise that vaccines expressing HCV structural proteins and containing the most conserved core and immunodominant E1/E2 could elicit highly cross-reactive and protective T cell immunity to various HCV genotypes. This may be crucial for elucidating the correlations between vaccine immunity and protection as well as for identifying the optimal design of candidate vaccines [1,6,7,11]. In this study, we developed a T cell-directed vaccine using replication-defective adenoviruses expressing HCV structural antigens.

Adenoviral vectors are attractive carriers for genetic vaccines because of their strong immunogenicity and their ability to transduce antigen-presenting cells (APCs) and elicit strong B and T cell immune responses to target antigens [20]. In mice and nonhuman primates, recombinant adenoviral 5 (rAd5) vector-based immunogens induce strong T cell responses toward a variety of target antigens [20-24]. Currently, several rAd5-based vaccines against a variety of infectious agents are in the preclinical and clinical stages of development [20-25]. However, pre-existing anti-Ad immunity can significantly dampen the response to the vaccine [20,21]. Previous reports have suggested that optimisation of delivery routes and regimens might overcome this limitation [20,21]. However, to the best of our knowledge, few studies have presented data on the immunity induced by rAd5-based HCV vaccines delivered via different routes and regimens.

No inexpensive animal model of HCV for vaccine evaluation currently exists [26]. One of the more easily available and more commonly used murine models is infection with a recombinant vaccinia virus encoding the protein of interest [23,24,27]. This surrogate model of HCV infection has been used to evaluate a variety of vaccine platforms, including a subunit protein, plasmid DNA, and human adenovirus vaccines [23,24,27].

In this study, we constructed an rAd5 vector expressing the HCV structural genes CE1E2 (rAd5-CE1E2) and a recombinant Tian Tan Vaccinia viral vector (rTTV) [28] expressing the same HCV target proteins (rTTV-CE1E2). We evaluated their immunogenicity via different delivery routes and regimens to optimise the immune response. Moreover, we characterised cross-protection in different groups of mice using a heterologous surrogate challenge assay based on a recombinant HCV (JFH1, 2a) vaccinia virus.

## Results

### Construction and expression of recombinant viral vector-based HCV vaccines

**Figure 1:**
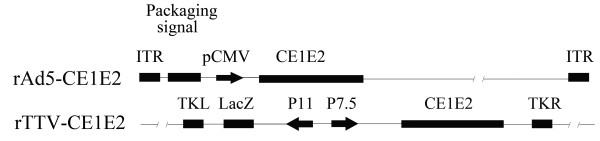
**Schematic representation of recombinant viral vectors encoding the HCV structural gene**. ITR, inverted terminal repeat; P7.5K, P7.5 later promoter; P11K, P11 later promoter; TKR, right thymidine kinase; TKL, left thymidine kinase.

**Figure 2:**
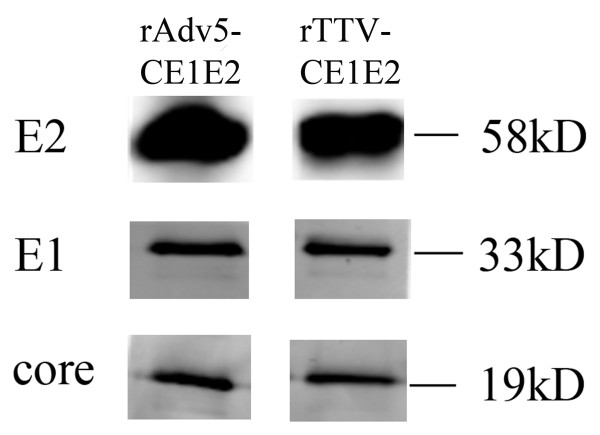
**Western blot analysis of HCV antigen expression of rAd5-CE1E2 and rTTV-CE1E2**. The expression of HCV antigen in HeLa cells infected with recombinant virus was detected by Western blot. Cell lysates were subjected to SDS-PAGE followed by immunoblotting with antibodies specific for the individual antigens, as described in the methods. The expression bands of E1, E2, and Core proteins and their molecular weights are indicated.

We constructed an rAd5-based vaccine (rAd5-CE1E2) expressing the HCV structural gene CE1E2 (Figure 1), as well as a recombinant vaccinia virus expressing the HCV structural gene CE1E2 (rTTV-CE1E2) (Figure 1). Expression of the target proteins was confirmed by Western blotting using a mouse mAb to the HCV core, E1, and E2 proteins (Figure 2A). All of the proteins were expressed as previously described in cell lysates infected with rAd5-CE1E2 or rTTV-CE1E2.

### Single vaccination with rAd5-CE1E2 induced significant CMI in mice

**Figure 3:**
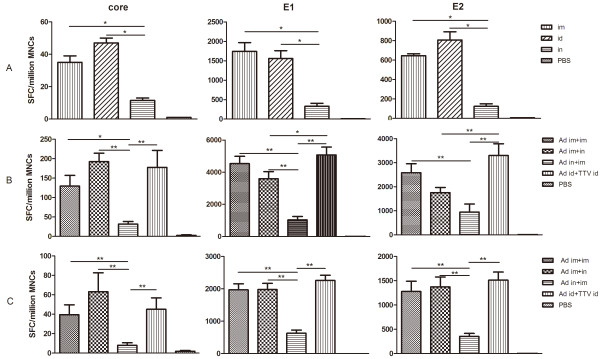
**Cellular immune responses in rAd5-CE1E2 immunised mice**. An ELISPOT assay of splenocytes after stimulation with an HCV peptide pool representing the core, E1, or E2 proteins. Splenocytes were isolated at weeks 2 (A, post-prime), 5 (B, post-boost), and 20 (C, long-term). The data are expressed as spot-forming cell (SFC) responses to different peptide pools, and are presented as means with SEM of six mice per group. Significant p values between the vaccinated groups are shown. Control group mice injected with PBS generated SFC responses of < 20 per 106 mononuclear cells. Significant p values between vaccinated groups are shown. * indicates p < 0.05, ** indicates p < 0.01.

To To compare the CMI response elicited by the various injection routes, we performed ELISPOT after a single vaccination. Splenocytes were harvested 2 weeks after immunisation and stimulated with an HCV peptide pool representing the core, E1, or E2 proteins. IFN-γ-positive spot-forming cells (SFCs) against the E1 or E2-2 peptide pools were detected in all immunised groups, whereas spots against the core peptide pool were detected in all groups. The cellular immune responses against E1 were much stronger than those against the core or E2 proteins. During E1 peptide pool screening we identified a single peptide, E1-21 (SQLFTFSPRRYETI), which had a high affinity. The group immunised via i.n. had significantly fewer SFCs than did the i.m. and i.d. groups; there were no significant differences between the i.m. and i.d. groups (Figure 3A).

### Prime-boost vaccination induces robust and sustained CMI in mice

To further compare the immunogenicity of rAd5-based HCV vaccines among different delivery routes and regimens, we used different vaccination combinations and monitored their effects via ELISPOT at 2 or 16 weeks post-boost. Of the prime-boost groups, the heterologous immune group (rAd5+rTTV) had more SFCs than the homologous immune groups (rAd5 only), and the i.n. priming group (rAd5 i.n.+i.m.) had fewer SFCs than the other groups (rAd5 i.m.+i.m., rAd5 i.m.+i.n.); these differences were statistically significant (Figure 3B). The CMI remained at a high level for a long period (over 16 weeks) in each of the prime-boost groups (Figure 3C), and the response level in each group was parallel to that detected (Figure 3B).

**Figure 4:**
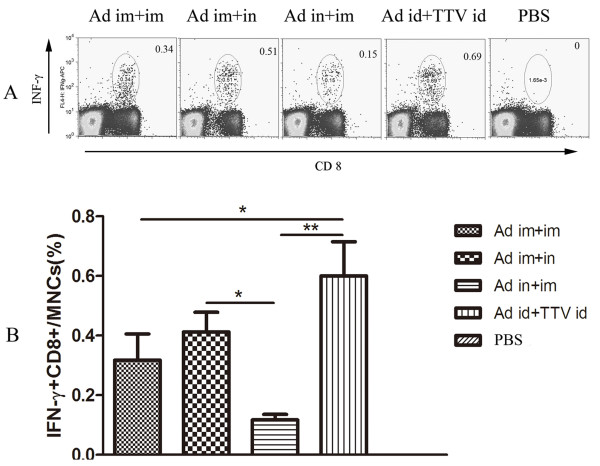
**CD8+ T-cell response obtained by ICS in rAd5-CE1E2 and rTTV-CE1E2 immunised mice**. Splenocytes isolated at week 20 were stimulated in vitro with peptide E1-21 (SQLFTFSPRRYETI) alone. Representative fluorescence-activated cell sorter flowgrams are shown in Figure 4A (percentages of IFN-γ+CD8+cells are shown in the right upper quadrants), and a summary of the percentages of IFN-γ-producing CD8+cells are presented in Figure 4B as the mean with SEM of four mice per group. Significant p values between the vaccinated groups are shown. * indicates p < 0.05, ** indicates p < 0.01.

To examine the CTL response more quantitatively, we performed ICS on the immunised mice. Splenocytes isolated at week 22 (16 weeks post-boost) were stimulated in vitro with the peptide E1-21. In Figure 4A, the number in the right-hand corner of each graph indicates the percentage of CD8+ cells that stained positive for IFN-γ. As shown in Figure 4B, the percentage of CD8+T cells that stained positive for IFN-γ in the heterologous immune group was higher than that in the other groups. Also, and similar to the ELISPOT results, among the homologous immune groups, the percentage of IFN-γ+CD8+T cells in the group primed via i.n. (Ad i.n.+i.m.) was significantly lower than in the other groups (rAd5 i.m.+i.m., rAd5 i.m.+i.n.).

### Humoral immune response in mice detected by ELISA

To assess the humoral immune response in mice induced by rAd5-CE1E2/rTTVCE1E2 vaccination, we monitored IgG antibody levels against the core, E1, and E2 proteins via ELISA (Table 1). The patterns of anti-E1 and anti-E2 antibody levels were identical to those of the T cell responses. Interestingly, and contrary to the T cell responses, the anti-core antibody titre of the i.n. priming group (Ad i.n.+i.m.) was markedly higher than the others, except for the heterologous prime-boost (rAd5 +rTTV) group. The humoral immune responses against HCV (IgG antibody anti-core/E1/E2) in the heterologous prime-boost (rAd5 +rTTV) group were much higher than those in the homologous immune groups (rAd5 i.m.+i.m., rAd5 i.m.+i.n.). Anti-adenoviral antibody levels were also evaluated by ELISA. IgG titres were > 8000 in the single-immunised groups and > 20000 in the prime-boost vaccination groups (Table 1). These data indicate that injections were successfully administered to each mouse.

**Table 1:** Humoral responses (anti-HCV IgG) in immunised groups detected by ELISA.

	IgG Antibody titer						
	
	Ad (i.m.)	Ad (i.d.)	Ad (i.n.)	Ad (i.m.+i.m.)	Ad (i.m.+i.n.)	Ad (i.n.+i.m.)	Ad (id)/TTV(id)
**Anti-core**	50	50	50	50	50	3200	400
**Anti-E1**	100	100	25	200	200	100	1600
**Anti-E2**	100	100	25	800	400	100	1600

Note: Antisera were collected 14 days post-immunization, and total IgG titres specific for the HCV antigen were determined by ELISA as described in the methods. Numbers represent titres of pooled sera from each group. Experiments were repeated at least three times and same results were obtained. The antibody titre was defined as the reciprocal of the serum dilution at which the absorbance (OD value) was twice that of sera in the non-immunized group.

### rAd5-HCV vaccination induced cross-protection in a surrogate challenge model

**Figure 5:**
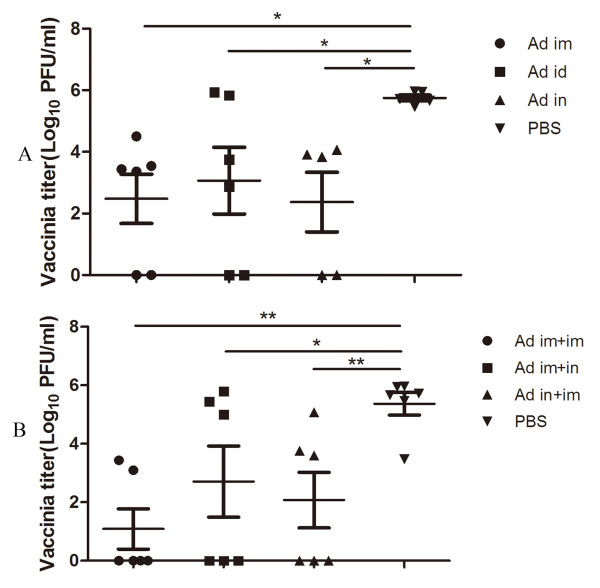
**Vaccinia titres in immunised mice challenged with rTTV-CE1E2**. Mice were sacrificed 5 days post-challenge. Vaccinia titres in ovaries were determined. Mice (five or six per group) received a single immunisation (Figure 5A). Mice (six per group) received two immunisations and were challenged after the second (booster) immunisation (Figure 5B). The data are presented as the mean with SEM of each group. Significant p values between the vaccinated groups are shown. * indicates p < 0.05, ** indicates p < 0.01.

To assess the effects of the immunity induced by rAd5-CE1E2 (1b), 1 × 107 pfu heterologous rTTV-JFH1(2a) was inoculated into immunised mice 8 weeks after the final injection. Mice were sacrificed 5 days after challenge. Then vaccinia titres in the ovaries were determined. As shown in Figure 5, the immunised groups were protected from heterologous challenge and showed significantly different responses than the control group. However, there were no significant differences among the immunised groups.

## Discussion

Development of a B-cell-based vaccine is difficult due to the high genetic variability of HCV [6-8]. A potent vaccine should induce a strong and long-term protective T cell response [6,7]. We constructed an rAd5-based and rTTV-based HCV vaccine expressing the HCV structural gene CE1E2 from HCV genotype 1b. We identified the antigenic composition of the vaccine vectors and evaluated their immunogenicity via different delivery routes and regimens. Our results indicate that those vaccines were able to elicit a CMI response against the HCV core, E1, and E2 antigens in mice. The response remained robust for an extended period (16 weeks post-vaccination). Induction of cross-protection by rAd5-based HCV CE1E2-induced responses were confirmed in vivo using a heterologous surrogate challenge assay based on a recombinant HCV (JFH1,2a) vaccinia virus.

Since it was discovered that T cells are important for clearance of HCV, a great deal of focus has been on developing vaccines that will induce T cell responses to HCV proteins [6,7]. A number of approaches, including the use of virus-like particles (VLPs) and defective or attenuated viral vectors with or without a prime-boost strategy, have been used to generate T cell responses against HCV antigens [6,7]. The use of HCV VLPs produced in insect cells has proven successful for inducing effective HCV-specific immune responses [29]. Interestingly, in these VLP studies, responses to the structural proteins were mainly specific to T cells with little or no neutralising antibody detected, and all responses resulted in modification of HCV infection and early control of replication following virus challenge [29]. In addition, a metaanalysis of HCV vaccine efficacy in chimpanzees indicated the importance of structural proteins that may activate T cell responses and thus mediate viral clearance [30]. We chose the structural proteins (1-746 amino acids [aa]) of the HCV isolates dominant in China (1b) as immunogens. This choice was also based on the fact that a similar structural gene (1-746 aa) can be assembled as a VLP when expressed in insect cells [29]. Western blot analysis indicated that the core, E1, and E2 proteins were expressed efficiently in cells infected with rAd5-CE1E2 vaccine.

The viral vectors used for HCV T cell vaccines, including adenovirus and vaccinia virus vectors, are common to a number of strategies used for other infectious agents (such as HIV and TB) [6,7,20-25,31,32]. A greater number of rAd5-based HCV vaccine candidates have successfully induced HCV-specific immune responses [23,24,31]. Interestingly, an adenovirus vector-based minigene vaccine encompassing the four domains of the HCV NS3, NS4, NS4A, and NS5B proteins that contain multiple class I/II restricted epitopes also induced strong and broad HCV specific T cell responses in HLA-A2 transgenic mice and may prove promising as a tool for inducing cross-reactive responses [31]. An extremely encouraging study reported that an rAd-based T cell vaccine expressing the NS3-NS4-NS5A-NS5B antigens elicited non-sterile, yet protective, immunity in four of five challenged chimpanzees [25]. Protection in this study was correlated with T cell responses, in particular with CD8+T cell-mediated immunity. Based on these reports, we selected rAd as a vector to carry the immunogen. In addition, an attenuated recombinant vaccinia virus (Tian Tan strain) was selected as a vector [33]. The data indicated that an rAd5-based HCV vaccine can elicit multi-antigen, robust, and long-lasting IFN-γ-producing CD8+T cell-mediated immunity in mice, with cross-protection. In addition, a heterologous rAd5/rTTV regimen elicited the strongest CMI and E1/E2-specific humoral immune response, compared to a homologous rAd5 regimen. These data are in accordance with previous studies of the T cell responses elicited by rAd-based HIV or HCV vaccines [20-22]. Despite the current controversies concerning the use of rAd-based vaccines for HIV-1, we demonstrated here that an rAd5-CE1E2-based T cell vaccine for HCV has significant cross-protective efficacy in our surrogate challenge model. The data in this proof-of-concept study have important implications for the application of novel T cell-based HCV vaccines.

The majority of rAd5-HCV vaccines have been tested in animal model via the i.m. or i.p. route. There are limited data in the literature comparing the immunogenicity and protection elicited by various rAd5-based HCV vaccine delivery routes and regimens. Thus, we assessed the humoral and cellular immune responses and cross-protection elicited in mice immunised via different delivery routes (i.m., i.n., i.d.) and regimens. The immune effects of each delivery route differed. Compared to the i.n. route, one injection of rAd-CE1E2 induced a stronger cellular immune response to the HCV structural gene when administered via i.m. or i.d. Similarly, priming via i.n. induced a lower IFN-γ T cell response than did i.m priming. These results demonstrate that the priming route may be an important determinant of immune effects. Of the two-injection groups, an rTTV-CE1E2 boost following rAd-CE1E2 priming induced the strongest T cell responses to the HCV core, E1, and E2 proteins. Similar to the cellular responses, the heterologous regimen induced the strongest antibody response to E1 and E2, while the homologous i.n.-primed group resulted in the lowest antibody levels. Curiously, for the anti-core antibody, the titre in the i.n.-primed homologous group was much higher than in the other groups. No significant neutralising antibody response was observed among the groups based on an HCVpp assay (data not shown). Both the antibody and CMI responses could be boosted further in our model system, because even the pre-existing anti-Ad5 IgG antibody levels in the immunised groups were high (Table 1).

The majority of vaccine studies have used a homologous virus challenge, i.e., the same genotype as the virus contained in the vaccine. In this study, we used a heterologous challenge model developed in our lab, which used a different subtype (2a) virus expressing an HCV-polyprotein with an approximately 18% genetic difference from the vaccine-based sequence (1b). We demonstrated in this model that vaccination of mice with an rAd5-based HCV CE1E2 vaccine strongly reduced the titre of rHCV-JFH1, and was able to fully protect immunised mice, although the i.n.-immunised and i.n.-primed regimens induced the weakest T cell responses. These results indicate that other mechanisms, such as antibody (core), are involved in protective immunity to HCV, not only the level of IFN-γ-producing T cells detected by ELISPOT or ICS. It remains possible, however, that other aspects of the CMI response may contribute to cross-protection of HCV vaccines [34]. In addition, the potential role of antibody-dependent cell-mediated virus inhibition (ADCVI) [35] should also be explored in a future study. Development of an immunocompetent small animal model would advance the field enormously [36], although care should always be taken in extrapolating data to humans, as many immune response studies in mice have not translated well to humans and other primates.

## Conclusions

We developed an HCV candidate vaccine based on an rAd5-vector and structural protein (CE1E2) immunogen. This vaccine elicits a potent and long-lasting boost during in vivo cross-reactive T cell-mediated immune responses with a single or double vaccination by different routes (i.n., i.m., i.d.) or combinations of routes (i.m.+i.m., i.m.+i.n., i.n.+i.m.). However, the humoral immune response (especially the neutralising antibody titre) was not strong. Our data support the idea that an rAd5-CE1E2-based HCV vaccine would show promise against HCV infection, and would be capable of inducing cross-protection. However, the protective potency was correlated not only with the IFN-γ SFC level, as detected by ELISPOT or ICS, it was correlated with other as-yet-unidentified factors that need to investigated further. There is still a great deal of missing information regarding the types of immune responses that correlate with protection or clearance of HCV after vaccination. New vaccine studies should also employ higher-level immunological analyses that examine the multifunctional activities of T cells and T cell phenotypes.

## Methods

### Generation of recombinant viruses

The HCV CE1E2 gene (1-746 aa HCV 1b subtype, Hebei isolate [37], NCBI accession no. L02836) was used to construct the rAd5-CE1E2 vector (Figure 1). The adenovirus type 5 construct is a prototypic first-generation vector containing an E1 deletion replaced with the HCV CE1E2 expression cassette. As the E1 deletion renders it replication-defective, the vector was propagated in HEK293 cells, which provided E1 in trans. The recombinant Ad5-virus was generated as reported previously [38]. The rAd5-CE1E2 vector was purified and amplified in HEK293 cells.

The original TTV strain and dual-promoter insertion vector pJSA1175 were produced in our laboratory [28,33]. The HCV CE1E2 gene was inserted into the SmaI site of the pJSA1175 vector. The rTTV vector was produced by transfection of pJSA1175-CE1E2 into CEF cells that were infected with TTV, and was designated rTTV-CE1E2 (Figure 1).

To obtain heterologous surrogate challenge viruses, the full-length ORF (1-3011 aa) gene from the HCV JFH1 (2a) strain was inserted into the SmaI site of the pJSA1175 vector. Insertion was followed by transfection of pJSA1175-JFH1 into CEF cells that had been infected by TTV. The resulting vector was designated rTTV-JFH1 (2a). The virus stock was purified and titrated as described previously [28,33].

### Characterisation of the immunogen

Expression of the target protein was identified by Western blot using a mouse mAb to the HCV core, E1, and E2 proteins. HeLa cells infected with recombinant virus were collected after 48 h, and processed by cell lysis (50 mM Tris pH 7.5, 70 mM β-mercaptoethanol). Lysates were then separated on a 10% or 15% polyacrylamide gel and transferred by electroblotting to a polyvinylidene fluoride (PVDF) membrane. The membrane was blocked for 1 h with 5% skim milk at 37°C and then incubated with monoclonal antibody (mAb) to the HCV core (ABR) or E1 (ABR) or E2 (AP33, from Dr. H. Patel) proteins overnight at 4°C. After being washed three times with phosphate-buffered saline (PBS) containing 0.5% Tween-20 (PBST), the membrane was protected from light and incubated with goat anti-mouse antibody (IRDye 800) for 1 h at 37°C. After washing three times in PBST, bands were detected using an infrared imaging system.

### Immunisation and challenge

Groups of female BalB/c mice were immunised at 6-8 weeks of age. They were divided into three groups and immunised once with rAd5-CE1E2 administered by intramuscular injection (i.m.), intranasal injection (i.n.), or intradermal injection (i.d.). The three homologous immune groups were then immunised with rAd5-CE1E2 as prime immunisation via i.m., i.m., or i.n., and boosted via i.m., i.n., or i.m., respectively, at 6-week intervals. Mice in one heterologous immunisation group were primed with rAd5-CE1E2 and boosted with rTTV-CE1E2 at 6-week intervals. In addition, mice immunised with PBS were used as controls. The viral doses were 5 × 109 vp/mouse for rAd5-CE1E2 or 1 × 107 pfu/mouse for rTTV-CE1E2.

Eight weeks after the last immunisation, mice were challenged with 1 × 107 pfu of rTTV-JFH1 (2a) by intraperitoneal injection (i.p.). They were sacrificed 5 days after challenge (peak vaccinia titre in ovaries), and their were ovaries harvested. After a freeze-thaw and homogenisation procedure, the vaccinia titre was determined by plaque assay using chicken embryo cells [28,31].

### Immune response analysis of vaccinated mice

A peptide library of HCV structural proteins (C/E1/E2), based on the Hebei isolate sequence of HCV genotype 1b, was synthesised as lengths of 13-17 aa with an overlap of 10 aa between fragments (ZhongKeYaGuan Co., Beijing, China). Peptides were dissolved in dimethyl sulfoxide (DMSO) at a concentration of 50 mg/ml. The core and E1 peptide pools contained 42 and 39 peptides, respectively. The E2 peptide pool contained 79 peptides separated into two pools: E2-1 (40 peptides) and E2-2 (39 peptides). The peptide pools were aliquoted, stored at -20°C, and used at a final concentration of 4 μg/ml.

### Enzyme-linked immunospot (ELISPOT) assays

Splenocytes were harvested at 2 weeks post-immunisation and stimulated with the HCV peptide pools. Briefly, multiscreen 96-well plates were coated overnight with 100 μl per well of 5 μg/ml anti-mouse gamma interferon antibody (IFN-γ) (BD Pharmingen) in PBS. Then the plates were washed three times with RPMI 1640 containing 10% FBS, blocked for 2 h with RPMI 1640 containing 10% FBS, and incubated with peptide pools and mononuclear spleen cells (MNCs) in triplicate in 100 μl reaction mixture volumes. The peptide pools used in this study spanned the HCV core, E1, and E2 proteins and comprised 13-17 aa peptides. Each peptide in a pool was present at a concentration of 4 μg/ml. Following incubation for 20-24 h at 37°C, cell suspensions were aspirated. The wells were washed twice with deionised (DI) water. Wells were allowed to soak for 3-5 min at each wash step. Then the wells were washed three times with 200 μl PBST before the addition of 100 μl detection antibody solution (BD Pharmingen). The lid was replaced and the plates were incubated for 2 h at room temperature. Then the wells were washed three times with 200 μl PBS, being allowed to soak for 1-2 min at each wash step. A prepared streptavidin-HRP solution was added at 100 μl per well and the plates were incubated for 1 h at room temperature, followed by four washes, and the addition of 100 μl prepared AEC substrate solution to each well. Spot development was monitored for 5-60 min. The reaction was stopped by washing with DI water. Plates were air-dried at room temperature and stored in a sealed plastic bag in the dark until analysis in an ELISPOT plate reader.

### Intracellular cytokine staining(ICS)

Splenocytes (2 × 106/sample) were cultured for 5 h at 37°C in 96-well round-bottom microtitre plates in 1640 supplemented with 10% FBS, and peptide E1-21 (SQLFTFSPRRYETI) and Brefeldin A (GolgiPlug; BD PharMingen) were added simultaneously. The E1-21 peptide was used at a concentration of 4 μg/ml. Control cells were incubated with an unrelated peptide or without any peptide. After washing, cells were incubated for 30 min at 4°C with 25 μl of a 1/100 dilution of a PE-labelled Ab to mouse CD8 and FITC-labelled Ab to mouse CD4 (BD PharMingen). The cells were washed again and permeabilised in 1 × Cytofix/Cytoperm (BD PharMingen) for 20 min at 4°C, washed three times with Perm/Wash (BD PharMingen), and then incubated in the same buffer for 30 min at 4°C with 50 μl of a 1/100 dilution of an APC-labelled Ab to mouse IFN-γ (BD PharMingen). After washing, the cells were examined by three-color flow cytometry.

### Enzyme-linked immunosorbent assay (ELISA)

Sera from animals were pooled, and diluted sera were applied to a 96-well plate previously coated with soluble E1 or E2 protein [39] for detection of anti-E1 or anti-E2 antibody, or coated with antigen containing the core and NS3 HCV fusion (Wantai Co., China) for anti-core antibody detection, or with recombinant adenovirus (mock) for anti-Ad antibody detection. Then the plates were blocked with skim milk and incubated for 1 h at 37°C. After washing with PBST, peroxidase-conjugated goat anti-mouse IgG was added and the plates were incubated for a further hour followed by a wash. A colour reaction was induced by adding 3,3,5,5-tetramethylbenzidine (TMB) peroxidase substrate solution. The reaction was stopped by adding 1 M H2SO4, and the absorbance was read at 450 nm. The antibody titre was defined as the reciprocal of the serum dilution at which the absorbance was twice that of sera of unimmunised mice.

### Data analysis

Significant differences between the experimental and control groups were evaluated using the one-way ANOVA analysis function in the SPSS software package (release 12.1; SPSS Inc., Chicago, IL). Differences were considered significant at p < 0.05.

### Ethical approval

According to the medical research regulation of Ministry of Health, China, this study was approved by the ethics committee of China CDC, which uses international guidelines to ensure confidentiality, anonymity, and informed consent.

## Competing interests

The authors declare that they have no competing interests.

## Authors' contributions

GJ and WB performed immunogenicity studies in mice and drafted the manuscript. DY and ZK generated viral constructs, and CH participated in the immunogenicity studies. WX and RL helped design the study. TW contributed ideas, directed the study, and analysed and interpreted the data. All authors read and approved the manuscript.
